# Supplementary material for: The Effects of Subjective Wellbeing and Self-Rated Health on Lifetime Risk of Cardiovascular Conditions in Women
Source: Int J Environ Res Public Health. 2023 Jul 17;20(14):6380. doi: 10.3390/ijerph20146380 (PMC10378917; doi:10.3390/ijerph20146380)
Supplement: Supplementary file 1 [file ijerph-20-06380-s001.zip › ijerph-2396680-supplementary.pdf]

Table S1. Descriptive statistics of characteristics at waves 1, 2, and 3 and its comparisons

Characteristics		Wave 1	Wave 2	Wave 3	Wave 1 vs 2 (p value)	Wave 1 vs 3 (p value)
**Age (years)**	**Mean (SD)**	45.73 (11.51)	54.65 (11.46)	63.74 (11.47)	<.001	<.001
**Income (US dollars)**	Median (IQR)	60,000 (32,500-92,500)	63,375 (31,250-87,500)	75,487 (35,000-88,375)	<.001	.407
**Race**	n(%)				<.001	<.001
White	n(%)	1607 (93.6%)	1581(92.1%)	1527 (89.0%)		
non-White	n(%)	109 (6.4%)	135 (7.9%)	189 (11%)		
Black		70 (4.1%)	65 (3.8%)	67 (3.9%)		
Native American	n(%)	4 (0.2%)	25 (1.5%)	20 (1.2%)		
Asian	n(%)	9 (0.5%)	11 (0.6%)	8 (0.5%)		
Other/multi racial	n(%)	26 (1.6%)	34 (2.0%)	94 (5.4%)		
**Education**					<.001	<.001
Less than high school	n(%)	105 (6.1%)	78 (4.5%)	100 (5.8%)		
High school diploma/GED	n(%)	486 (28.3%)	469 (27.3%)	446 (26.0%)		
Some college	n(%)	543 (31.6%)	539 (31.4%)	538 (31.4%)		
College degree or higher	n(%)	582 (33.9%)	630 (36.7%)	632 (36.8%)		
**Marital status**	n(%)				<.001	<.001
Married	n(%)	1210 (70.5%)	1166 (67.9%)	1036 (60.4%)		
Not married	n(%)	506 (29.5%)	550 (32.1%)	680 (39.6%)		
**Employment status**	n(%)				<.001	<.001
Currently working	n(%)	1106 (64.5%)	923 (53.8%)	697 (40.6%)		
Not currently working	n(%)	610 (35.5%)	793 (46.2%)	1019 (59.4%)		
**Smoking status**	n(%)				<.001	<.001
Current	n(%)	297 (17.3%)	240 (14%)	173 (10.1%)		
Former/never	n(%)	1419 (82.7%)	1476 (86%)	1543 (89.9%)		
**Diabetes/high blood sugar**					<.001	<.001
Yes	n(%)	34 (2.0%)	110 (6.4%)	182 (10.6%)		
No	n(%)	1682 (98%)	1606 (93.6%)	1534 (89.4%)		
**Pre-existing CVD**					<.001	<.001
Yes	n(%)	386 (22.5%)	718 (41.8%)	933 (54.4%)		
No	n(%)	1330 (77.5%)	998 (58.2%)	783 (45.6%)		
**Life satisfaction**	**Mean (SD)**	7.81 (1.22)	7.85 (1.19)	7.80 (1.31)	.516	.599
**Positive affect**	**Mean (SD)**	3.39 (0.73)	3.42 (0.72)	3.44 (0.71)	.118	.021
**Negative affect**	**Mean (SD)**	1.55 (0.63)	1.53 (0.58)	1.50 (0.60)	.263	.001
**Physical self-rated health**	**Mean (SD)**	3.64 (0.92)	2.35 (0.95)	2.58 (1.04)	<.001	<.001
**Health compared to others**	**Mean (SD)**	2.28 (0.89)	2.23 (0.96)	2.25 (1.00)	.022	.287

Abbreviations. SD = Standard Deviation; IQR = Inter quartile range; CVD = cardiovascular disease. 1. P-values are from chi-square test for categorical variables and t-test for continuous variables.

1. Overall, differences in demographic characteristics between waves 1 and 2 reflect 10 years of the aging process, as well as wave 1 vs wave 3 (17-19 year) differences. Participants at waves 2 and 3 were 10 and about 19 years older, respectively. Participants income and education levels increased, rates of being married and smokers declined, and diabetes and CVD rates increased. While life satisfaction did not change over the 3 waves, positive affect increased from waves 2 to 3, and negative affect and self-reported health levels declined over time. Self-reported health compared to others declined in the first 10 years and then flattened afterwards.
2. Self-reports of race and ethnicity showed discrepancies over the time, with less individuals self-identifying as white over the 20 years. Some participants changed self identified race to non-whites from whites over the 20 years.

Table S2. Correlations for Life satisfaction, positive affect, and negative affect

	Life Satisfaction	Negative Affect	Positive Affect	VIF
Life Satisfaction	1.00	---	---	1.62
Negative Affect	-.50 (p<.001)	1.00	---	1.83
Positive Affect	.56 (p<.001)	-.64 (p<.001)	1.00	1.96

Abbreviation. VIF = Variance Information Factor

1. VIF >1 indicates multicollinearity and 10 is considered as high.

Table S3. Logistic regression analysis for subjective well-being and self-rated health (wave 2) predicting cardiovascular conditions 10 years later at wave 3.

Variables at Wave 2	CVD Conditions (Wave3) OR (95% CI)		
	Model 1	Model 2	Model 3
**Subjective Well-Being Model**			
Age	**1.04 (1.03, 1.05)**	**1.04 (1.03, 1.06)**	**1.04 (1.06, 1.08)**
White vs non-White	.80 (.45, 1.40)	.82 (.47, 1.44)	.85 (.48, 1.50)
Not married vs married	.91 (.67, 1.22)	.96 (.71, 1.31)	.97 (.71, 1.32)
< HS degree	---	---	---
HS degree	.80 (.44, 1.44)	.79 (.43, 1.44)	.82 (.45, 1.50)
Some college	1.01 (.57, 1.83)	1.01 (.55, 1.84)	1.07 (.58, 1.95)
4 year college or >	.79 (.43, 1.44)	.80 (.43, .147)	.86 (.46, 1.59)
Not working vs working	1.05 (.79, 1.38)	1.05 (.80, 1.39)	1.07 (.81, 1.41)
Income (US dollars)	1.00 (1.00, 1.00)	1.00 (1.00, 1.00)	1.00 (1.00, 1.00)
Current vs Former/never smoker	1.24 (.88, 1.76)	1.21 (.86, 1.56)	1.14 (.85, 1.54)
Diabetic vs non-diabetic	2.82 (.86, 9.24)	2.82 (1.21, 9.29)	3.39 (1.23, 9.40)
Pre-existing CVD vs no CVD	**17.97 (13.33, 24.23)**	**17.06 (12.64, 23.02)**	**17.18 (12.72, 23.21)**
Life satisfaction		**.83 (.74, .93)**	**.83 (.72, .95)**
Positive affect			1.17 (.91, 1.50)
Negative affect			1.30 (.96, 1.75)
C-statistic	.84	.85	.85
AIC	1496.24	1487.57	1488.49
**Self-Rated Health Model**			
Age	**1.04 (1.03, 1.05)**	**1.04 (1.03, 1.05)**	**1.05 (1.03, 1.06)**
White vs non-White	.89 (.52, 1.49)	.97 (.57, 1.63)	.96 (.57, 1.62)
Not married vs married	.98 (.73, 1.30)	.96 (.72, 1.28)	.94 (.70, 1.25)
< HS degree	---	---	---
HS degree	.90 (.51, 1.58)	.95 (.54, 1.68)	.95 (.53, 1.68)
Some college	1.03 (.59, 1.83)	1.16 (.65, 2.06)	1.18 (.67, 2.10)
4 year college or >	.82 (.46, 1.46)	.96 (.53, 1.71)	.96 (.53, 1.72)
Not working vs working	1.02 (.78, 1.32)	1.05 (.81, 1.37)	1.05 (.80, 1.37)
Income (US dollars)	1.00 (1.00, 1.00)	1.00 (1.00, 1.00)	1.00 (1.00, 1.00)
Current vs Former/never smoker	1.17 (.84, 1.63)	1.07 (.77, 1.50)	1.07 (.76, 1.50)
Diabetic vs non-diabetic	2.49 (.82, 7.54)	2.08 (.68, 6.39)	2.20 (.70, 6.89)
Pre-existing CVD vs no CVD	**16.23 (12.28, 21.45)**	**14.21 (10.71, 18.86)**	**13.87 (10.44, 18.43)**
Physical SRH		**.72 (.63, .84)**	**.83 (.70, .98)**
Health compared to others			**1.30 (1.11, 1.53)**
C-statistic	.84	.85	.85
AIC	1655.04	1631.68	1629.02

Abbreviations. HS = high school; CVD = cardiovascular disease; SRH = self-rated health; AIC = Akaike Information Criteria

1. It is statistically significant at $\alpha = 0.05$ significance level when 95% CI does not contain 1.

Table S4. Logistic regression analysis, odds ratios (95% confidence intervals), for subjective well-being and self-rated health (wave 1) at baseline predicting cardiovascular conditions 10 years (wave 2) and 19 years later (wave 3) exclusion of pre-existing CVD.

Variables at Wave 1	Cardiovascular conditions (wave 2) OR (95% CI)			Cardiovascular conditions (wave 3) OR (95% CI)		
	Model 1	Model 2	Model 3	Model 1	Model 2	Model 3
**Subjective Well-Being Model**						
Age	1.06 (1.05, 1.07)	1.07 (1.06, 1.08)	1.07 (1.06, 1.08)	1.06 (1.05, 1.07)	1.07 (1.06, 1.08)	1.07 (1.06, 1.08)
White vs non-white	.45 (.30, .69)	.46 (.30, .70)	.46 (.30, .70)	.56 (.35, .88)	.59 (.37, .93)	.61 (.38, .97)
Married vs not married	.96 (.75, 1.22)	1.04 (.81, 1.33)	1.04 (.81, 1.33)	.93 (.72, 1.19)	1.03 (.79, 1.33)	1.02 (.79, 1.33)
< HS degree	---	---	---	---	---	---
HS degree	.61 (.39, .96)	.63 (.40, .99)	.63 (.40, .99)	.63 (.39, 1.03)	.65 (.39, 1.06)	.66 (.40, 1.09)
Some college	.68 (.43, 1.07)	.69 (.44, 1.08)	.69 (.44, 1.09)	.78 (.47, 1.28)	.82 (.49, 1.35)	.85 (.51, 1.41)
4 year college or >	.50 (.32, .80)	.51 (.32, .81)	.51 (.32, .81)	.56 (.34, .92)	.60 (.36, .99)	.63 (.38, 1.04)
Working vs not working	1.04 (.83, 1.29)	1.04 (.83, 1.30)	1.04 (.83, 1.31)	1.09 (.86, 1.37)	1.11 (.88, 1.41)	1.13 (.90, 1.44)
Income (US $)	1.00 (1.00, 1.00)	1.00 (1.00, 1.00)	1.00 (1.00, 1.00)	1.00 (1.00, 1.00)	1.00 (1.00, 1.00)	1.00 (1.00, 1.00)
Current smoker vs former/never	1.19 (.90, 1.57)	1.13 (.85, 1.49)	1.12 (.85, 1.49)	1.24 (.92, 1.66)	1.16 (.86, 1.56)	1.14 (.85, 1.54)
Diabetic vs not diabetic	2.82 (1.28, 6.22)	2.50 (1.12, 5.58)	2.50 (1.12, 5.57)	3.79 (1.40, 10.31)	3.36 (1.21, 9.29)	3.39 (1.23, 9.40)
Life satisfaction		.80 (.74, .88)	.81 (.73, .91)		.73 (.66, .81)	.76 (.67, .85)
Positive affect			.98 (.81, 1.20)			1.06 (.86, 1.31)
Negative affect			1.02 (.82, 1.27)			1.25 (.97, 1.62)
C-statistic	.70	.71	.71	.70	.72	.72
AIC	2126.43	2120.66	2124.52	2149.51	1961.09	1953.78
**Self-Rated Health Model**						
Age	1.06 (1.05, 1.07)	1.06 (1.05, 1.07)	1.07 (1.06, 1.08)	1.06 (1.04, 1.07)	1.06 (1.05, 1.07)	1.07 (1.06, 1.08)
White vs non-white	.46 (.30, .69)	.55 (.36, .84)	.52 (.34, .80)	.59 (.38, .90)	.71 (.46, 1.11)	.69 (.44, 1.07)
Married vs not married	.96 (.75, 1.23)	.99 (.77, 1.27)	.97 (.75, 1.24)	.95 (.74, 1.21)	.98 (.77, 1.25)	.97 (.75, 1.24)
< HS degree	---	---	---	---	---	---
HS degree	.61 (.39, .96)	.74 (.47, 1.18)	.71 (.45, 1.14)	.70 (.43, 1.12)	.85 (.52, 1.38)	.83 (.51, 1.35)
Some college	.68 (.43, 1.07)	.88 (.55, 1.41)	.86 (.54, 1.37)	.81 (.50, 1.30)	1.05 (.65, 1.72)	1.04 (.63, 1.69)
4 year college or >	.50 (.32, .80)	.68 (.42, 1.10)	.66 (.41, 1.07)	.58 (.36, .95)	.79 (.48, 1.30)	.78 (.47, 1.29)
Working vs not working	1.04 (.83, 1.29)	1.12 (.89, 1.40)	1.13 (.90, 1.42)	1.03 (.82, 1.28)	1.09 (.87, 1.37)	1.10 (.88, 1.38)
Income (US $)	1.00 (1.00, 1.00)	1.00 (1.00, 1.00)	1.00 (1.00, 1.00)	1.00 (1.00, 1.00)	1.00 (1.00, 1.00)	1.00 (1.00, 1.00)
Current smoker vs former/never	1.19 (.90, 1.57)	1.09 (.82, 1.45)	1.09 (.82, 1.44)	1.22 (.92, 1.61)	1.12 (.85, 1.49)	1.12 (.84, 1.48)
Diabetic vs not diabetic	2.82 (1.28, 6.22)	2.02 (.88, 4.60)	1.93 (.84, 4.46)	3.27 (1.29, .8.29)	2.42 (.92, 6.34)	2.36 (.89, 6.25)
Physical SRH		.61 (.54, .69)	.67 (.59, .78)		.61 (.58, .69)	.66 (.57, .76)

Health compared to others			1.22 (1.06, 1.41)			1.16 (1.01, 1.34)
C-statistic	.70	.73	.74	.70	.73	.73
AIC	2126.43	2074.61	2070.56	2149.51	2102.34	2104.06

Abbreviations. HS = high school; SRH = Self-rated health; AIC = Akaike Information Criteria.

1. It is statistically significant at α=0.05 significance level when 95% CI does not contain 1.
